# Targeting MYC for the treatment of breast cancer: use of the novel MYC-GSPT1 degrader, GT19630

**DOI:** 10.1007/s10637-024-01504-5

**Published:** 2025-01-28

**Authors:** Minhong Tang, John Crown, Michael J. Duffy

**Affiliations:** 1 UCD School of Medicine, Conway Institute of Biomolecular and Biomedical Research, University College Dublin, Dublin, Ireland; 2 Department of Medical Oncology, St Vincent’s University Hospital, Dublin, Ireland; 3 Clinical Research Centre, St Vincent’s University Hospital, Dublin, D04T6F4 Ireland

**Keywords:** MYC, Cancer, Molecular glue, Treatment, Breast cancer, GT19630

## Abstract

**Background:**

Since MYC is one of the most frequently altered driver genes involved in cancer formation, it is a potential target for new anti-cancer therapies. Historically, however, MYC has proved difficult to target due to the absence of a suitable crevice for binding potential low molecular weight drugs.

**Objective:**

The aim of this study was to evaluate a novel molecular glue, dubbed GT19630, which degrades both MYC and GSPT1, for the treatment of breast cancer.

**Methods:**

The antiproliferative potential of GT19630 was evaluated in 14 breast cancer cell lines representing the main molecular subtypes of breast cancer. In addition, we also investigated the effects of GT19630 on apoptosis, cell cycle progression, cell migration, and degradation of the negative immune checkpoint protein, B7-H3.

**Results:**

GT19630 inhibited cell proliferation, blocked cell cycle progression, promoted apoptosis, and decreased cell migration at low nanomolar concentrations in breast cancer cell lines. By contrast, previously described MYC inhibitors such as specific MYC-MAX antagonists affected these processes at micromolar concentrations. Consistent with the ability of MYC to promote immune evasion, we also found that GT19630 degraded the negative immune checkpoint inhibitor, B7-H3.

**Conclusions:**

We conclude that the novel molecular glue, GT19630, is a potent mediator of endpoints associated with cancer formation/progression. Its ability to degrade B7-H3 suggests that GT19630 may also promote host immunity against cancer. To progress GT19630 as a therapy for breast cancer, our finding should now be confirmed in an animal model system.

**Supplementary Information:**

The online version contains supplementary material available at 10.1007/s10637-024-01504-5.

## Introduction

Overall, relatively good progress has been made in the treatment of breast cancer in recent years, especially in patients with estrogen receptor (ER)-positive/progesterone receptor (PR)-positive and HER-2-positive disease [1]. Despite this progress, breast cancer remains a major cause of cancer-related mortality in women, worldwide [1, 2]. To further improve outcome, especially in patients lacking ER, PR, or HER2, i.e., those with triple-negative (TN) disease, new therapies are required [3].

One of the most frequently altered genes in breast cancer is amplification and overexpression of the *c-MYC* oncogene (hereafter referred to as *MYC*) [4]. Indeed, *MYC* is the most frequently amplified gene in breast cancer [5–7], although other mechanisms are also likely to contribute to its increased expression [8, 9]. In breast cancer, amplification or overexpression of *MYC* has been associated with poor prognosis [10, 11] and resistance to a variety of systemic therapies including endocrine therapy [12], chemotherapy [13], CDK4/6 inhibitors [14], mTOR inhibitors [15], and immunotherapy [16–18]. Due to its high frequency of alterations in breast malignancy, its causative role in tumorigenesis/tumor progression and its role in conferring resistance to diverse therapies, MYC is a highly attractive target for new anti-cancer therapies for this disease [19–21].

Although an attractive target, developing drugs to inhibit MYC has historically been challenging due to the absence of a readily identifiable crevice for high-affinity binding of low molecular weight anti-cancer compounds (19–21). Furthermore, the predominant localization of MYC in the nucleus limits the use of large molecule treatments such as the traditional therapeutic monoclonal antibodies or monoclonal antibody-conjugates. Despite these historical difficulties, a small number of promising inhibitors for MYC-dependent cancers have recently been described [19–21], some of which have entered clinical trials (e.g., OMO-103, MRT-2359, WBC100) [22–25]. No MYC inhibitor, however, has been approved for clinical use.

One of the most promising emerging strategies for targeting difficult-to-drug anti-cancer targets involves the use of protein degraders such as PROTACs (proteolysis targeting chimeras) or molecular glues [26, 27]. PROTACs are heterobifunctional molecules containing 3 separate components, i.e., a ligand for binding to a specific target protein (e.g., MYC), a ligand that recruits an E3 ubiquitin ligase, and a linker that connects these 2 ligands [26, 27]. Molecular glues, on the other hand, enable the direct interaction between an E3 ubiquitin ligase and its specific target. Theoretically, PROTACs/molecular glues should be able to degrade and eliminate any protein target, thus offering the potential to target cancer-driver proteins that historically were difficult to inhibit such as MYC. The aim of this study was to investigate a novel molecular glue that degrades MYC and GSPT1 known as GT19630, as a potential new treatment for breast cancer.

## Materials and methods

### Discovery and preliminary studies with GT19630

The cereblon-based molecular glue, GT19630, was discovered using a structural activity relationship (SAR) strategy for MYC degraders in MYC-driven HL60 AML cells [28, 29]. Evidence of GT19630 binding to MYC was established using MYC pull-down experiments with biotin-labelled GT19630. Importantly, the binding of biotin-labelled GT19630 by MYC was inhibited by a non-biotin-labelled compound. This finding indicates a direct interaction between GT19630 and MYC. Subsequent analyses revealed that in addition to degrading MYC, GT19630 also degraded the translation termination protein, GSPT1 (G1 to S phase transition proteins 1) [28, 29].

### Materials

GT19630 [28] and GT19077 [30] were gifts from Kintor Pharmaceuticals Limited (Kintor), China, while MYCMI-6 [31, 32] was a gift from Prof. Lars-Gunnar Larsson, Karolinska Institutet, Stockholm, Sweden. MYCi975 (HY-12960) [33] was purchased from MedChemTronica, Sweden, the Annexin-V-FITC Apoptosis Detection Kit from Invitrogen, the apoptosis proteins antibody arrays from Abcam (Human Apoptosis Array, ab134001), and the immune-associated proteins arrays from Raybiotech (Human Immune Checkpoint Array C1, AAH-ICM-1–4).

### Cell culture, cell viability assays, and apoptosis assays

The breast cancer cell lines used were cultured and maintained as previously described [34, 35]. Similarly, cell viability was evaluated, as previously reported [34, 35]. In brief, cells were treated with a series of GT19630 concentrations ranging from 0 to 100 nM. For measuring apoptosis, cell lines were seeded in 6-well plates at a density of 2 × 10^5^ per well and incubated overnight at 37 °C. Cells were then treated with dimethyl sulfoxide (DMSO) as control or the indicated concentrations of GT19630. Following incubation for 48 h, cells were harvested and stained with annexin-V and propidium iodide using the Annexin-V-FITC Apoptosis Detection Kit. FACS analysis was performed using a BD FACSCanto II instrument.

### Western blot analysis

Extracted proteins (70 or 120 µg) were separated on a 10% handmade SDS-PAGE and transferred onto polyvinylidene difluoride or nitrocellulose membranes (Cytiva) [35]. Membranes were blocked in 5% milk TBST for 1 h at room temperature which was followed by incubation with the primary antibody, i.e., for MYC (Abcam, ab32072), for XIAP (Santa-Cruz Biotech, sc-55550) or for B7H3 (Santa-Cruz Biotech, sc-376769). GAPDH (Merck Millipore) was used as a loading control. After washing 3 times (10 min each time) in TBST, membranes were immersed in 5% milk TBST containing a secondary antibody (Santa-Cruz Biotech) for 1.5 h at room temperature, followed by another 3 washes. Bands were developed with super signal chemiluminescence (ECL) (Thermo Fisher Scientific) and visualized using a LI-COR instrument. Protein intensities were quantified by densitometry using ImageJ software.

### Cell cycle analysis

Cells treated with GT19630 for 48 h were harvested and fixed by adding drop by drop 3 mL of ice-cold 70% ethanol (diluted with PBS). Fixed cells were stained with FxCycle™ PI/RNase Staining Solution (Thermo-Fisher) and subjected to cell cycle analysis using a BD FACSCanto II flow cytometry machine, according to manufacturer’s protocol. Fluorescence was measured using a 488 nM excitation source and a 576 nM emission filter. Cell cycle phase percentages were calculated using FlowJo software.

### Protein screening using membrane antibody arrays

Expression of apoptotic proteins was analyzed using the Abcam Human Apoptosis Array (ab134001) and immune-associated proteins by the Raybiotech Human Immune Checkpoint Array C1 (AAH-ICM-1–4), following the manufacturer’s guidelines. Briefly, protein was extracted from BT549 and CAMA1 cells following treatment for 48 h with 3 nM GT19630 or vehicle control, using the provided lysis buffer. Membranes were blocked and incubated with protein samples overnight at 4 °C. After washing with the provided buffers, membranes were incubated overnight at 4 °C with detection antibody and then incubated at room temperature with HRP-Streptavidin for 2 h. Following a final wash, membranes were incubated with a detection buffer and imaged using a LI-COR detector. ImageJ software was used to generate densitometry values which were then normalized to the internal controls on the membrane.

### Cell migration

BT549, CAMA1, and MDA-MB-468 cells were treated with DMSO control or 3 nM GT19630 for 24 h. Cells were then harvested and seeded in Transwell chambers (24-well, 8.0-µm pore) (Fisher Scientific, Ireland), according to the manufacturer’s protocol. Briefly, 1 × 10^5^ cells per mL in 400 µl serum-free medium were seeded in the upper chamber while 600 µL of complete medium was added to the lower chamber as a chemoattractant. After incubation for 10–16 h at 37 °C, cells were fixed with 4% paraformaldehyde, made permeable with 1% Triton X-100, and stained with 0.1% crystal violet solution. Cells that migrated to the lower surface of the polycarbonate membrane were visualized using a Leica DFC295 Digital Color camera. To measure the extent of decreased cell migration, the images were divided into 8 equal parts. Four parts were randomly selected, and the number of cells located in each of these 4 areas was counted. This count was taken to represent the total number of cells in the whole image.

### RNA extraction and real-time PCR (qPCR)

RNA was extracted from cell lines using a TRIzol reagent (Sigma-Aldrich). One µg of RNA was reverse transcribed into cDNA using the High-Capacity cDNA Reverse Transcription Kit (Thermo Fisher Scientific). B7H3 and XIAP primers were supplied by Eurofins Genomics. GAPDH (Eurofins Genomics) was used as a reference control. The amplification process was performed with the Roche Light Cycler 480, according to the manufacturer’s instructions.

### Statistical analysis

All experiments were performed at least 3 times. Raw data were analyzed using Microsoft Excel 2021. GraphPad Prism 5 was used to graph the calculated data points and calculate statistical values. The significance of data was evaluated using Student’s unpaired, two-tailed *t*-test. A *p*-value of < 0.05 was deemed statistically significant.

## Results

### Effect of GT19630 on degradation of MYC in breast cancer cell lines

**Fig. 1:**
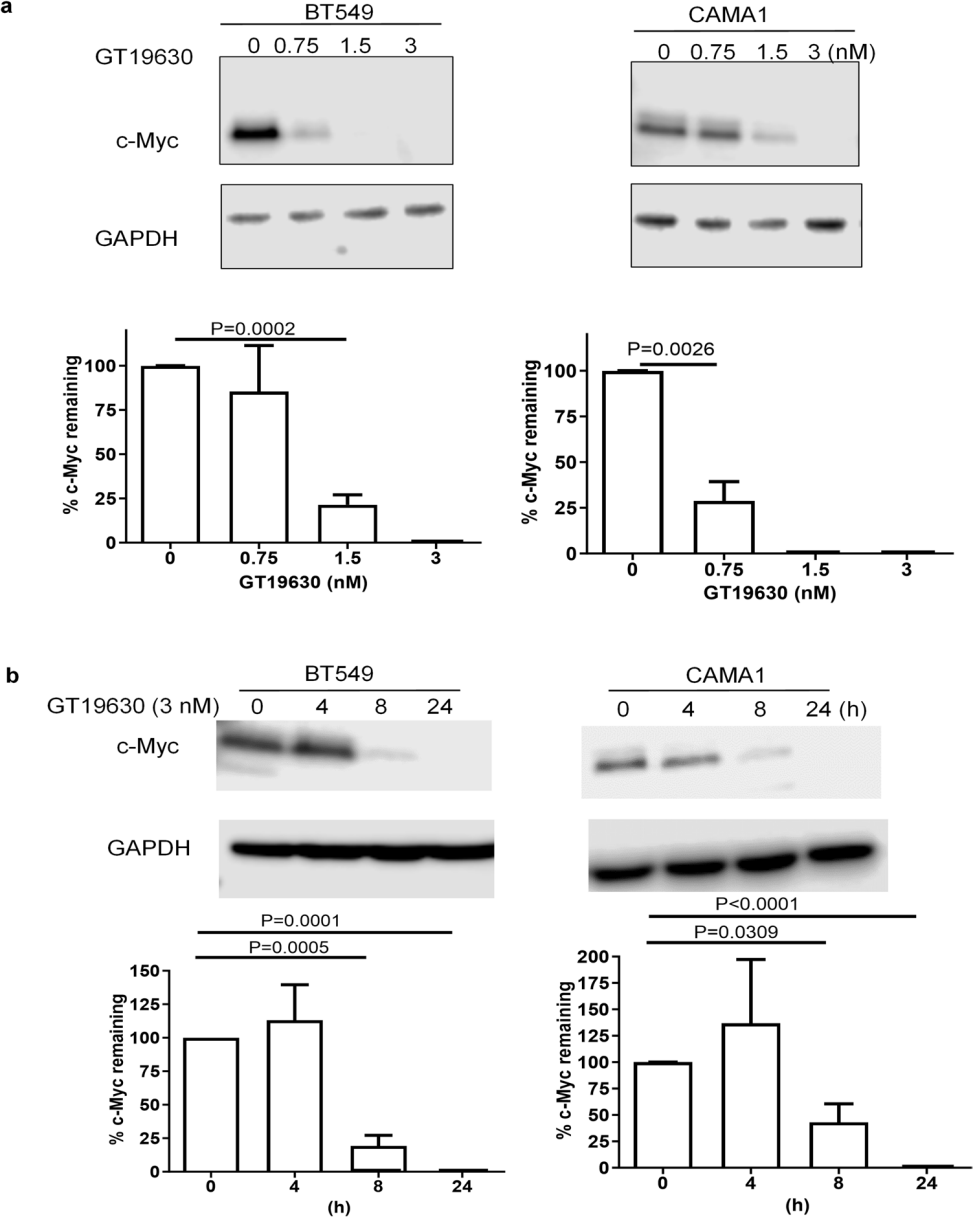
Effect of GT19630 on degradation of MYC. **a** Effect of varying concentration of GT19630 on MYC degradation in BT549 and CAMA1 cell lines. Cells were treated with the indicated concentrations of GT19630 for 48 h. **b** Time course of degradation of MYC in BT549 and CAM1 cell lines. Cells were treated with 3 nM of GT19630 for the indicated times. Amount of MYC protein remaining following treatment was calculated as a percentage of DMSO control. Data plotted are means ± S.E.M (*n* = 3). Statistical significance was evaluated using Student’s unpaired two-tailed *t*-test

To investigate the degradative potential of GT19630 against MYC, we tested the molecular glue’s ability to eliminate the oncoprotein in 2 breast cancer cell lines, i.e., BT-549 and CAMA1. Compared with MYC-MAX antagonists such as MYCi975 and MYCMI-6 which have previously been reported to degrade MYC at µM concentrations [32, 35], GT19630 degraded the oncoprotein at low nM concentrations (Fig. 1a). Indeed, degradation began to occur with concentrations as low as 0.75 nM in BT549 cells and at 1.5 nM in CAMA1 cells. As regards the time course of degradation, the process began as early as approximately 8 h following initiation of treatment in both cell lines and appeared to be complete by 24 h; at least no visible bands were detected by 24 h (Fig. 1b).

### Effect of GT19630 on the proliferation of human breast cancer cell lines

**Fig. 2:**
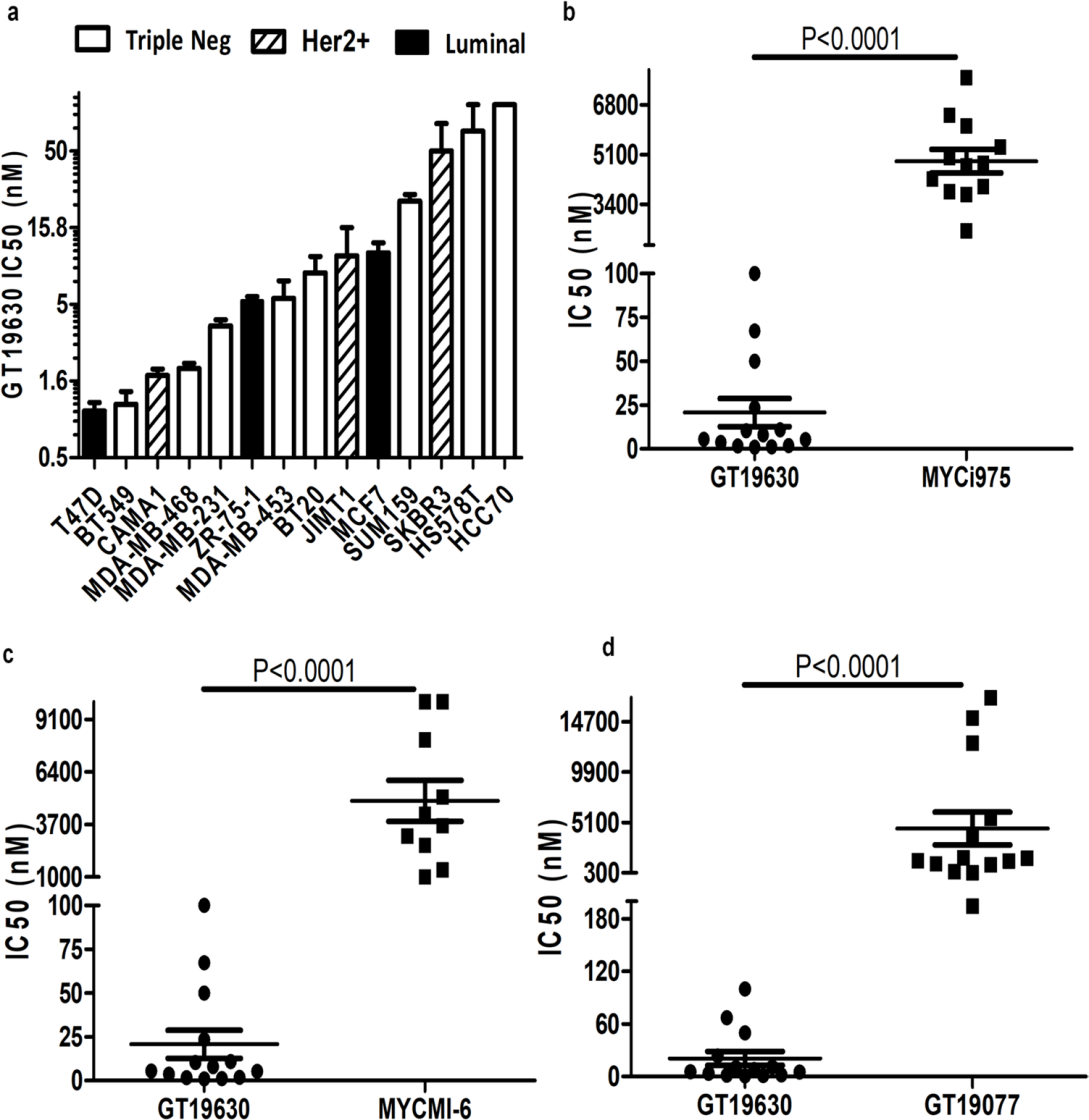
Effect of GT19630 on inhibition of cell proliferation. **a** IC50 values for the anti-proliferative effect of GT19630 on 14 breast cancer cell lines. Cells were treated with a concentration of GT19630 ranging from 0 to 100 nM. After 5 days of incubation, the extent of inhibition of cell growth was detected by the MTT assay. IC50 values were calculated and graphed using GraphPad Prism 5. **b** Scatter plot showing the relationship between IC50 values for GT19630 and MYCi975. **c** Scatter plot displaying the relationship between IC50 values for GT19630 and MYCMI-6. **d** Scatter plot showing the relationship between IC50 values for GT19630 and GT19077. Data plotted are means ± S.E.M (*n* = 3). Statistical significance was evaluated using Student’s unpaired two-tailed *t*-test

The effect of GT19630 on inhibition of cell growth was explored using a panel of 14 human breast cancer cell lines. Following 5 days of incubation with GT19630, the IC50 values (half-maximal inhibitory concentration) across the panel of cell lines used were found to vary from approximately 1 to approximately 100 nM (Fig. 2a). Of note, the IC50 values for GT19630 were significantly lower than those for 3,2 previously described MYC-MAX antagonists, MYCi975 [35], MYCMI-6 [32] or GT19077 [30] (*p* < 0.0001 for all comparisons) (Fig. 2b–d). Thus, GT19630 was approximately one order of magnitude more sensitive for inhibition of cell proliferation than the previously used MYC-MAX antagonists. No significant correlation, however, was found between the IC50 values for GT19630 and any of the MYC-MAX antagonists (Suppl. Figure 1a–c).

To establish if GT19630 had a preferential effect on cell lines from any of the specific molecular subtypes of breast cancer, we compared the IC50 values in ER-positive (luminal), HER2-positive, and TN breast cancer cell lines. Although the number of cell lines from the different subtypes was relatively small, GT19630 appeared to have similar anti-proliferative activity across the subtypes (Suppl Fig. 1d). Thus, GT19630 may have broad anti-proliferative activity in breast cancer, irrespective of the molecular subform of the disease.

**Fig. 3:**
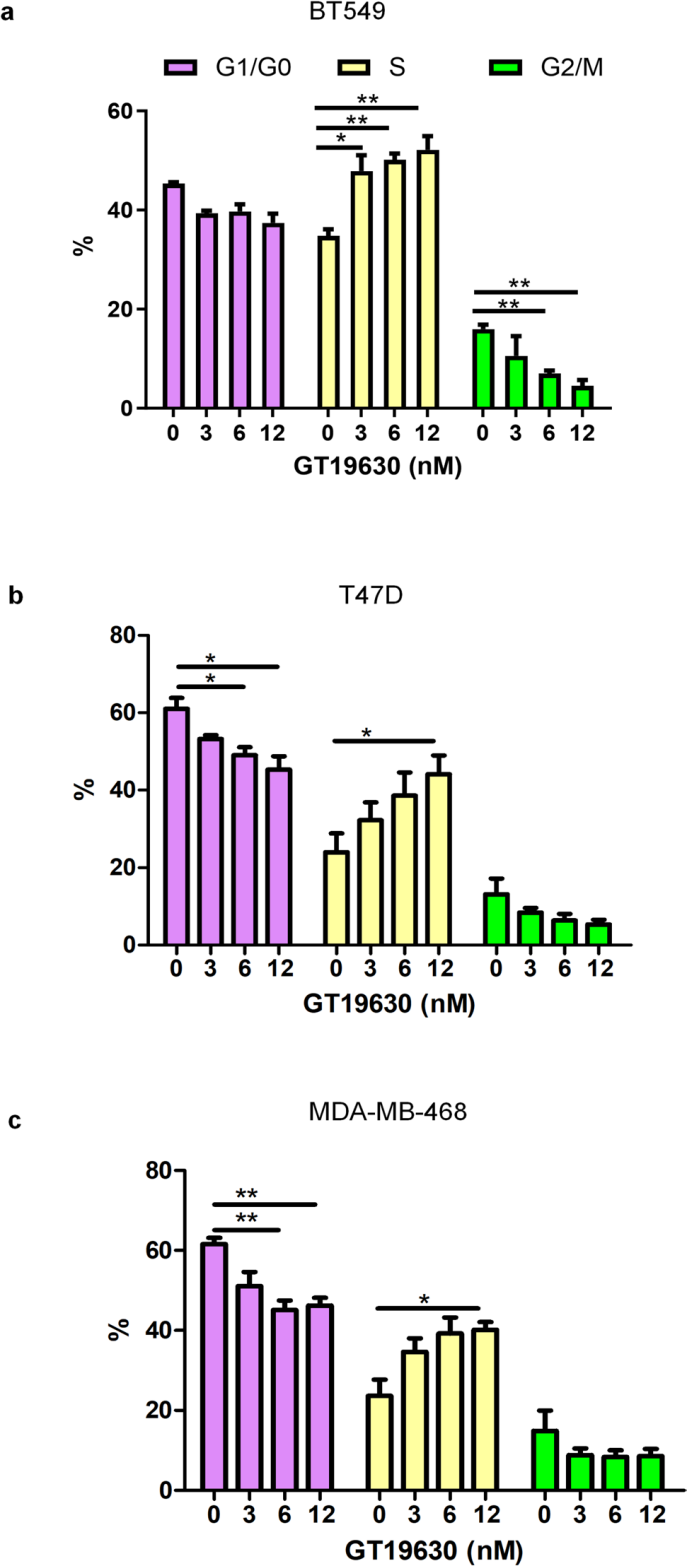
Effect of GT19630 on cell cycle arrest. Cell cycle analysis was performed by flow cytometry following treatment of cell lines for 48 h with the indicated concentrations of GT19630, **a** BT549, **b** T47D, and **c** MDA-MB-468. % of the cell cycle in each cell cycle phase was graphed and calculated using FlowJo software. Data were graphed using GraphPad Prism 5. Results shown are means ± S.E.M (*n* = 3). Statistical significance was evaluated using Student’s unpaired two-tailed *t*-test

To further study the anti-proliferative effects of GT19630, we performed cell cycle analysis on 3 different cell lines following treatment with the compound. As shown in Figs. 3a–c and Suppl. Figure 2a–c, treatment with GT19630 for 48 h resulted in the accumulation of cells at the S phase of the cell cycle, whereas the proportion of cells at the G1/G0-phase and G2/M-phases was decreased.

### Effect of GT19630 on the induction of apoptosis

**Fig. 4:**
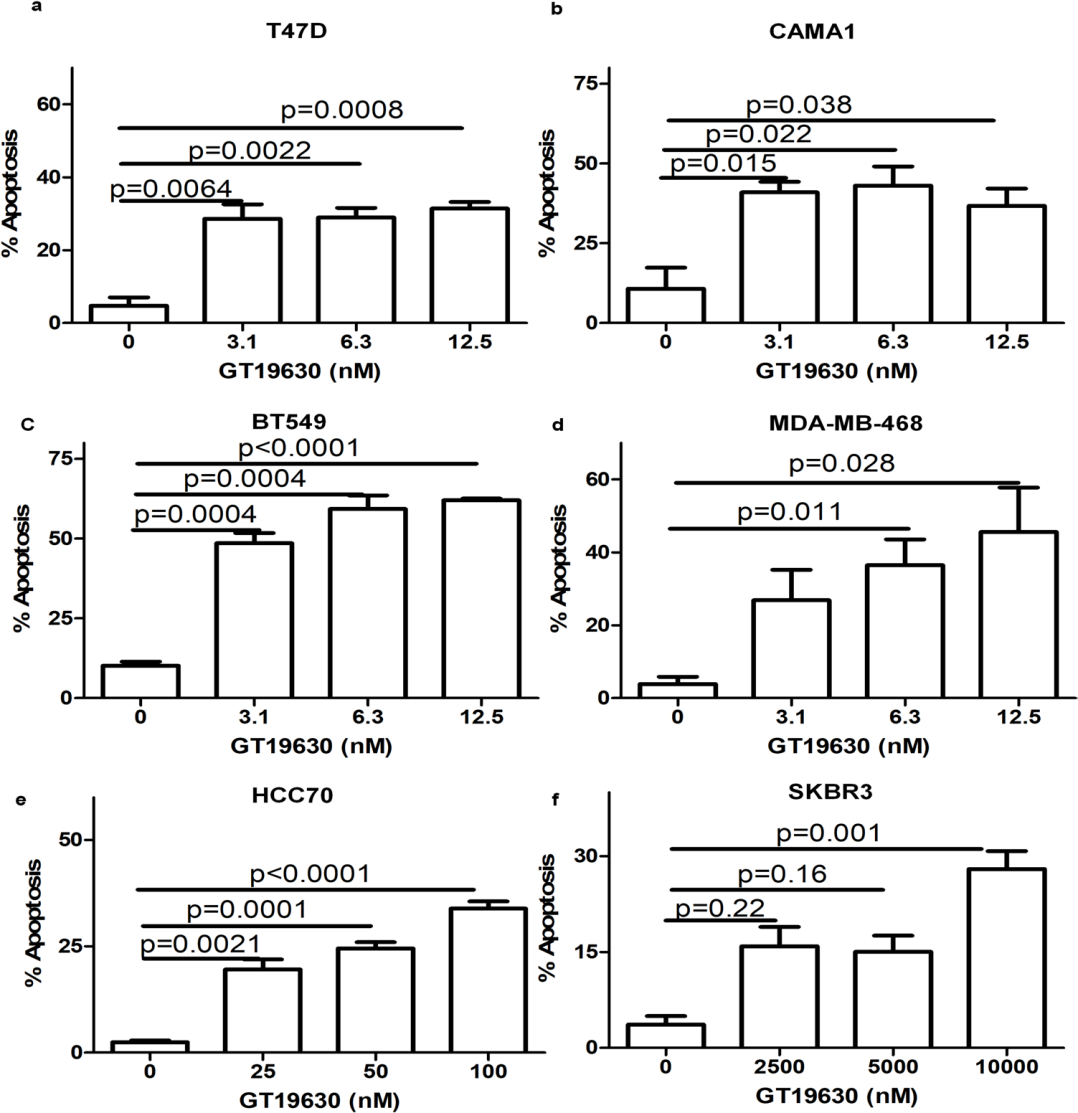
Effect of GT19630 on apoptosis. Apoptosis was measured by flow cytometry, following 48 h incubation with the indicated concentrations of GT19630, **a** T47D, **b** CAMA1, **c** BT549, **d** MDA-MB-468, **e** HCC70, and **f** SKBR3 cells. % induction of apoptosis was calculated and graphed using GraphPad Prism 5. Data plotted are means ± S.E.M (*n* = 3). Statistical significance was evaluated using Student’s unpaired two-tailed *t*-test

To establish if the GT19630-mediated growth inhibition resulted from induction of cell death, we investigated the potential ability of the molecular glue to induce apoptosis in 6 cell lines. Although GT19630 induced apoptosis in all the cell lines tested, the extent of the process was dependent on the cell line investigated, the concentration of GT19630 used, and the time of incubation (Fig. 4a–f). Interestingly, the 2 cell lines most resistant to apoptosis, i.e., HCC70 and SKBR3, tended to have relatively high IC50 values for inhibition of proliferation (IC50 values > 40 nM). In contrast, the cell lines more susceptible to induction of apoptosis, i.e., T47D, CAMA1, BT549, and MDA-MB-468 all had IC50 values < 5 nM. These findings suggest that there was a trend between high levels of apoptosis induction and low IC50 values, i.e., increased sensitivity to inhibition of proliferation.

**Fig. 5:**
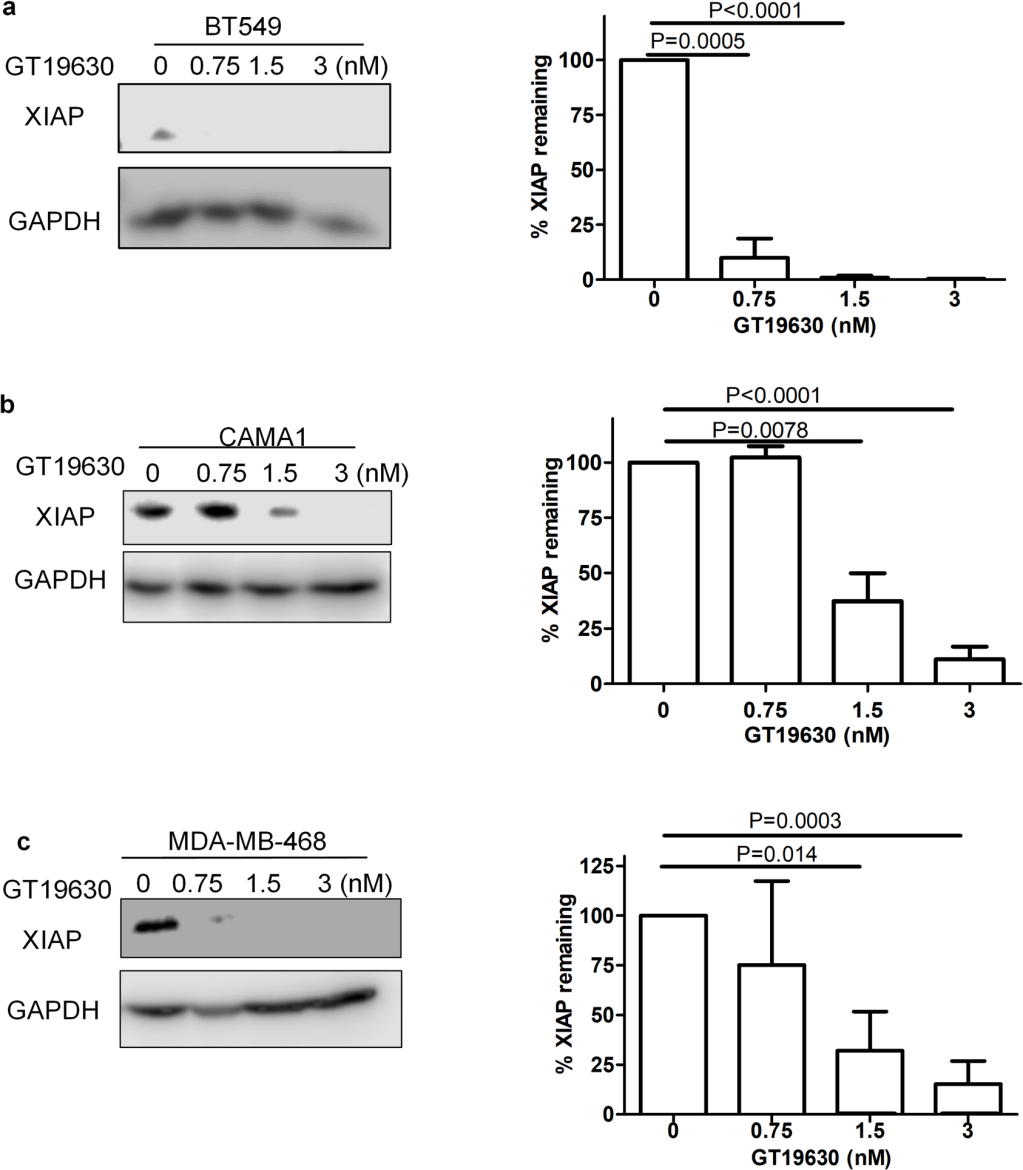
Effect of GT19630 on degradation of XIAP. **a** BT549, **b** CAMA1, and **c** MDA-MB-468 cells were incubated with the indicated concentrations of GT19630 for 48 h. Harvested cell lysates were subjected to Western blotting and stained using an antibody against XIAP (Santa-Cruz Biotech, sc-55550). GAPDH was used as a loading control. Data were calculated and graphed using GraphPad Prism 5. Values are means ± S.E.M (*n* = 3). Statistical significance was evaluated using Student’s unpaired two-tailed *t*-test

To investigate the possible mechanism(s) of apoptosis induced by GT19630, we initially studied the effect of the molecular glue on 43 apoptosis-associated proteins using membrane antibody arrays. As shown in Fig. Suppl. 3a, b, one of the proteins downregulated in both the BT549 and CAMA1 cell lines was the inhibitor of apoptosis protein (IAP), XIAP. To confirm these findings, we incubated 3 breast cancer cell lines, BT549, CAMA1, and MDA-MB-468, with different concentrations of GT19630 and measured XIAP by qPCR or Western blotting. As seen in Fig. 5, treatments with GT19630 resulted in decreased protein expression levels of XIAP in all 3 cell lines. To establish if the downregulation of XIAP occurred at the transcriptional level, we investigated the effect of GT19630 on XIAP mRNA expression in 3 cell lines. As shown in Fig. Suppl 3c, GT19630 had no effect on XIAP mRNA in 2 of the cell lines. However, the degrader appeared to increase XIAP mRNA expression in the MDA-MB-468 cell line. As XIAP is a potent anti-apoptotic protein [36], its downregulation may, at least in part, be responsible for mediating the GT19630-induced apoptosis. Interestingly, we previously reported that the MYC-MAX antagonist, MYCi975, decreased another member of the IAP family of apoptosis inhibitors, i.e., survivin [35].

### Effect of GT19630 on immune checkpoint proteins

**Fig. 6:**
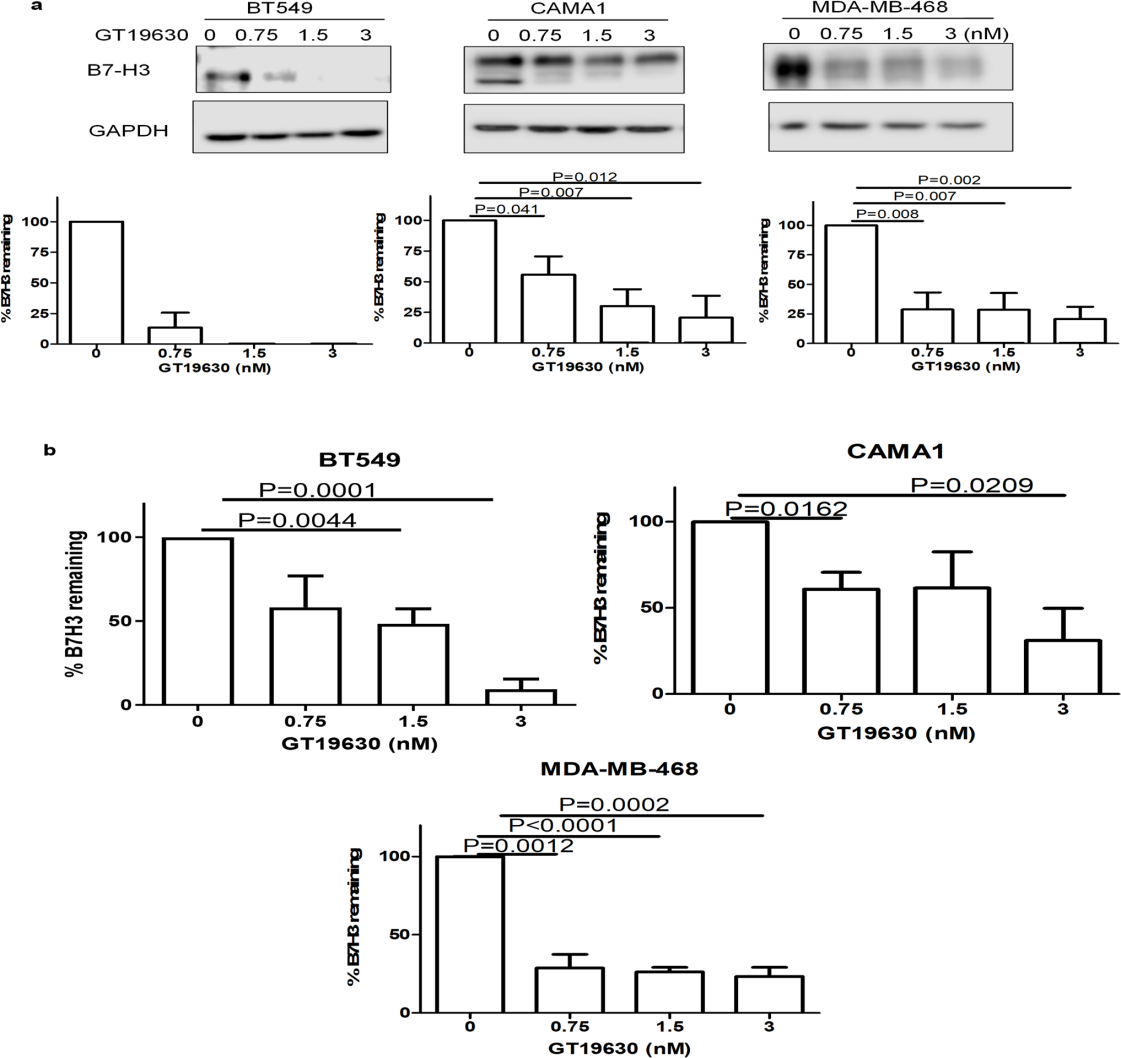
Effect of GT19630 on the degradation of the immune checkpoint protein, B7-H3. BT549, CAMA1, and MDA-MB-468 cells were incubated with the indicated concentrations of GT19630. Harvested cell lysates were assessed **a** by Western blotting using an anti-B7H3 antibody (Santa-Cruz Biotech, sc-376769). GAPDH was used as the loading control and **b** ELISA using B7H3 Elisa kit human B7-H3 DuoSet ELISA kits (R&D Systems, DY1949-05). Expression fold changes and % of B7-H3 protein remaining were calculated and graphed using GraphPad Prism 5

One of the mechanisms by which MYC promotes cancer formation/progression is by promoting immune evasion [37]. MYC inhibitors might be expected to reverse this process and thus promote immune responsiveness. To investigate if GT19630 might alter immune responsiveness, we tested its effect on a panel of immune regulatory proteins using membrane antibody arrays. As shown in Suppl. Figure 4a, b, only 9 of the 23 immune regulatory proteins on the array were detected in the BT549 and CAMA1 cells investigated. One of the proteins downregulated in both cell lines was the inhibitory immune checkpoint protein, B7-H3 (encoded by *CD276*) [37]. To confirm this downregulation, we performed both Western blotting and ELISA for B7-H3 following treatment of BT549, MDA-MB-468, and CAMA1 cells with 3 nM GT19630. As shown by both these measurements (Fig. 6a, b), GT19630 decreased protein expression levels of B7-H3 in all the 3 cell lines tested but did not affect mRNA expression levels (Fig. Suppl. 4c).

### Effect of GT19630 on cell migration

**Fig. 7:**
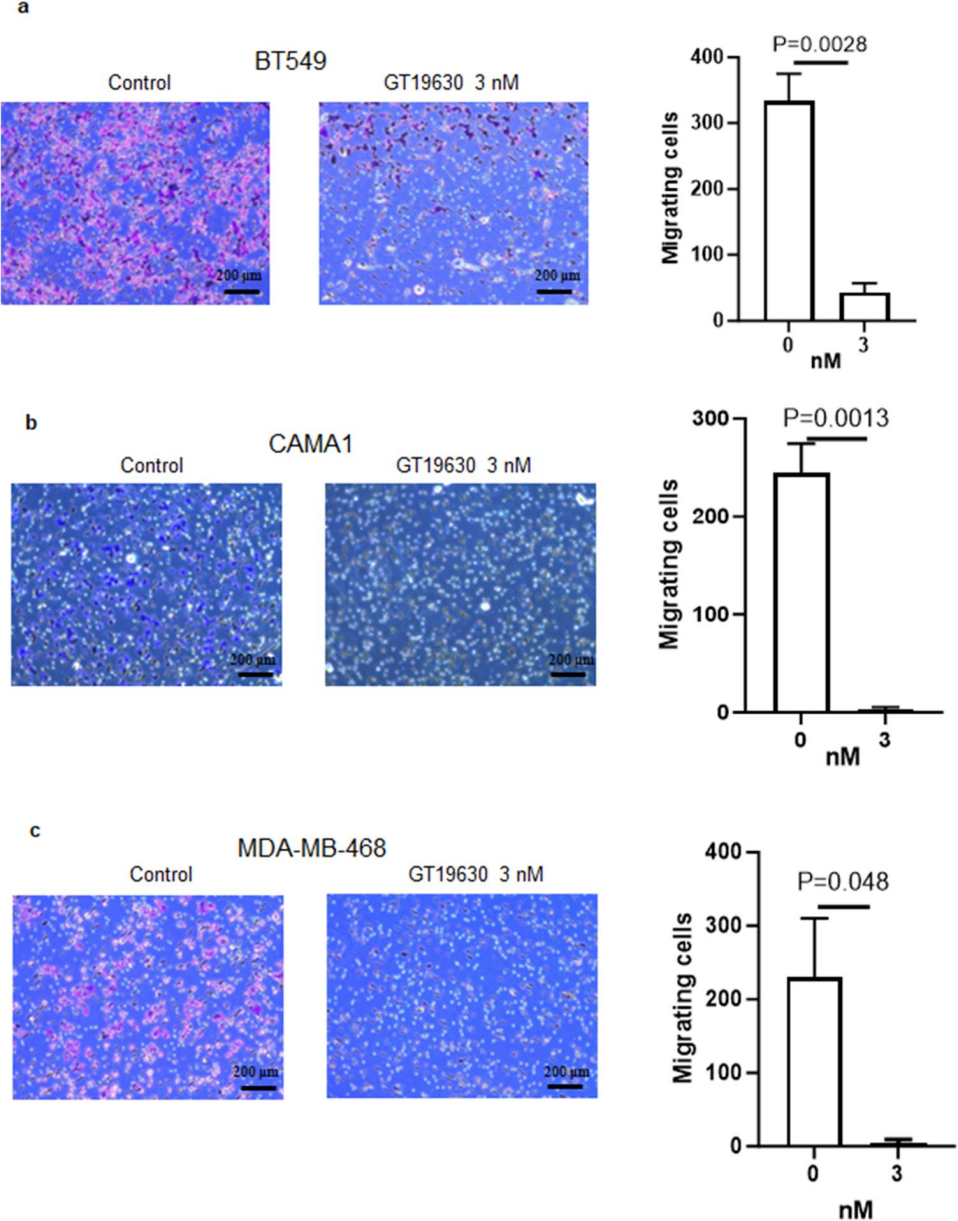
Effect of GT19630 on cell migration. BT549 (**a**), CAMA1 (**b**), and MDA-MB-468 (**c**) cells were treated with DMSO or 3 nM of GT19630 for 24 h. Migration was determined using Transwell assays. The number of migrating cells was counted and graphed using GraphPad Prism 5. Data plotted are means ± S.E.M (*n* = 3). Statistical significance was evaluated using Student’s unpaired two-tailed *t*-test. Scale bar = 200 µm

Tumor cell migration followed by metastasis represents the main cause of death in patients with cancer. To determine if GT19630 altered cell migration, we used Transwell assays. As shown in Fig. 7a–c, treatment with the molecular glue significantly reduced cell migration in all 3 cell lines investigated. It could be argued that the reduced migration was due to GT19630-induced cell death by apoptosis. However, as shown in Suppl. Figure 5, there was no significant induction of apoptosis over the time course of our migration assay, indicating that the decreased migration was not due to cell death by apoptosis.

## Discussion

Although previous studies showed that specific MYC inhibitors had anticancer potential in breast cancer experimental models [32, 35, 38], this, to our knowledge, is one of the first to describe in detail the use of a protein degrader against the oncoprotein. Compared with other types of small molecule anticancer compounds, molecular glues/PROTACS have several advantages [26, 27]. One of these is that protein degraders act in a catalytic manner, meaning that a single degrading molecule can eliminate multiple protein copies of a drug target. This in turn may allow the use of low or substoichiometric concentrations of the degrader. In contrast, most standard small molecule inhibitors act in an occupancy-driven manner that requires continuous and relatively high local concentrations to be effective. Another advantage of molecular glues/PROTACS is that these compounds can theoretically totally eliminate target proteins, whereas other types of small molecule inhibitors usually fail to effect complete inhibition/elimination [26, 27]. This potential ability to eliminate all of a specific target should be of particular value for mutated cancer driver proteins but perhaps not for MYC (see below).

Here, we show that treatment of breast cancer cell lines with the molecular glue, GT19630, degraded MYC, inhibited cell proliferation, blocked cell cycle progression, induced apoptosis, and decreased cell migration. Thus, GT19630 negated several processes associated with cancer progression. Compared with previously described MYC inhibitors such as MYC-MAX antagonists which usually require µM concentrations to be effective [32, 35], all these endpoints were achieved with low nM concentrations of GT19630, at least in vitro. Potentially, this ability to act at low concentrations could minimize toxicity, should GT19630 progress to a clinical trial. As discussed above, this ability to act at low concentration may at least partly relate to the catalytic nature of the molecular glue.

Alternatively, effectiveness at low concentrations may be due to the dual ability of GT19630 to degrade GSPT1 (involved in the termination of protein translation) as well as MYC [28, 29]. Indeed, several cereblon-related molecular glues/PROTACS that degrade GSPT1 have been reported to possess independent cytotoxicity activity with some having progressed to clinical trials [39–42]. Indeed, one of the selective GSPT1 degraders, known as MRT-2359 (Monte Rosa), was found to exhibit superior activity against MYC-dependent cancers than against cancers expressing low levels of MYC. MRT-2359 is currently undergoing a phase I/II, open-label, multicenter clinical trial in patients with specific previously treated solid tumors [24]. Preliminary results from the trial presented in a press release reported good tolerability, pharmacokinetic, and pharmacodynamics properties in heavily pre-treated patients with lung tumors or high-grade neuroendocrine cancer. Importantly, administration of MRT-2359 was claimed to decrease tumor size and induce partial responses in some heavily pretreated patients (https://menafn.com/1107255455/Monte-Rosa-Therapeutics-Announces-Interim-PKPD-And-Clinical-Data-For-MRT-2359-In-Phase-12-Trial-For-MYC-Driven-Solid-Tumors) [24]. Clearly, these promising findings require confirmation in larger studies and publication in peer-reviewed journals.

As mentioned above, in addition to its direct effects on cancer cells, one of the mechanisms by which MYC plays a role in cancer formation is by promoting immune evasion [37]. Consequently, inhibition or elimination of MYC might be expected to reverse this evasion and promote immune responsiveness. Consistent with this hypothesis, we found that GT19630 degraded the negative immune checkpoint protein, B7-H3. B7-H3 is a member of the B7 family of immune regulators; the best known of which is B7-H1 (better known as PD-L1) [43]. Like B7-H1, B7-H3 is also involved in immunosuppression [43]. Importantly, PD-L1 is the target for several clinically approved immune checkpoint inhibitors that are in everyday use [44]. Based on the success of anti-PD-L1 therapies, several trials using monoclonal antibodies against B7-H3 have recently commenced [43]. The ability of a small molecule such as GT19630 to degrade B7-H3 may have similar therapeutic value to B7-H3 monoclonal antibodies such as reversing immune suppression and promoting an anti-tumor immune response. Thus, the ability to degrade B7-H3 could potentially further enhance the anticancer activity of GT19630 in vivo*.*

A potential problem with our work described here as well as with several articles involving molecular glues or PROTACS is that the substrate specificity of the degrader is rarely investigated in detail. As mentioned above, we do, however, know that GT19630 in addition to degrading MYC can also proteolyze GSPT1. However, in most studies including ours, the full range of proteins, other than the primary target, potentially degraded by the molecular glue/PROTAC, is not known and indeed would be difficult to comprehensively investigate.

In summary, we have shown that GT19630 exerts multiple activities in breast cancer cell lines, including inhibition of proliferation, promotion of apoptosis, decreased cell migration, and suppression of the negative immune checkpoint protein, B7-H3. All these findings when taken together suggest that GT19630 should have anti-cancer activity in vivo. A word of caution, however—it would be important to avoid total elimination of MYC should GT19630 or related MYC degraders enter clinical trials. This is because MYC is involved in multiple normal biological processes. Consequently, total degradation could lead to serious side effects. Thus, the dose of the degrader administered will have to be carefully selected and monitored.

Our cell line data should now be confirmed in appropriate animal models in which both efficacy and toxicity will be evaluated. Although preliminary findings suggest that GT19630 exhibits favorable pharmacokinetic properties and apparently to be non-toxic in the animal models investigated to date [28, 29], these findings require further confirmation with longer-term follow-up. With such confirmation as well as the demonstration of efficacy, GT19630 should be considered for investigation in clinical trials.

## Supplementary Information

Below is the link to the electronic supplementary material.

ESM 1 (PNG 264 KB )

Supplementary file1 (TIF 291 KB)

ESM 2 (PNG 285 KB )

Supplementary file2 (TIF 1040 KB)

ESM 3 (PNG 463 KB )

Supplementary file3 (TIF 1509 KB)

ESM 4 (PNG 307 KB )

Supplementary file4 (TIF 985 KB)

ESM 5 (PNG 140 KB )

Supplementary file5 (TIF 234 KB)

## Data Availability

No datasets were generated or analysed during the current study.
