# Supplementary figures and images for: Targeting MYC for the treatment of breast cancer: use of the novel MYC-GSPT1 degrader, GT19630
Source: Invest New Drugs. 2025 Jan 28;43(1):167–79. doi: 10.1007/s10637-024-01504-5 (PMC11868176; doi:10.1007/s10637-024-01504-5)

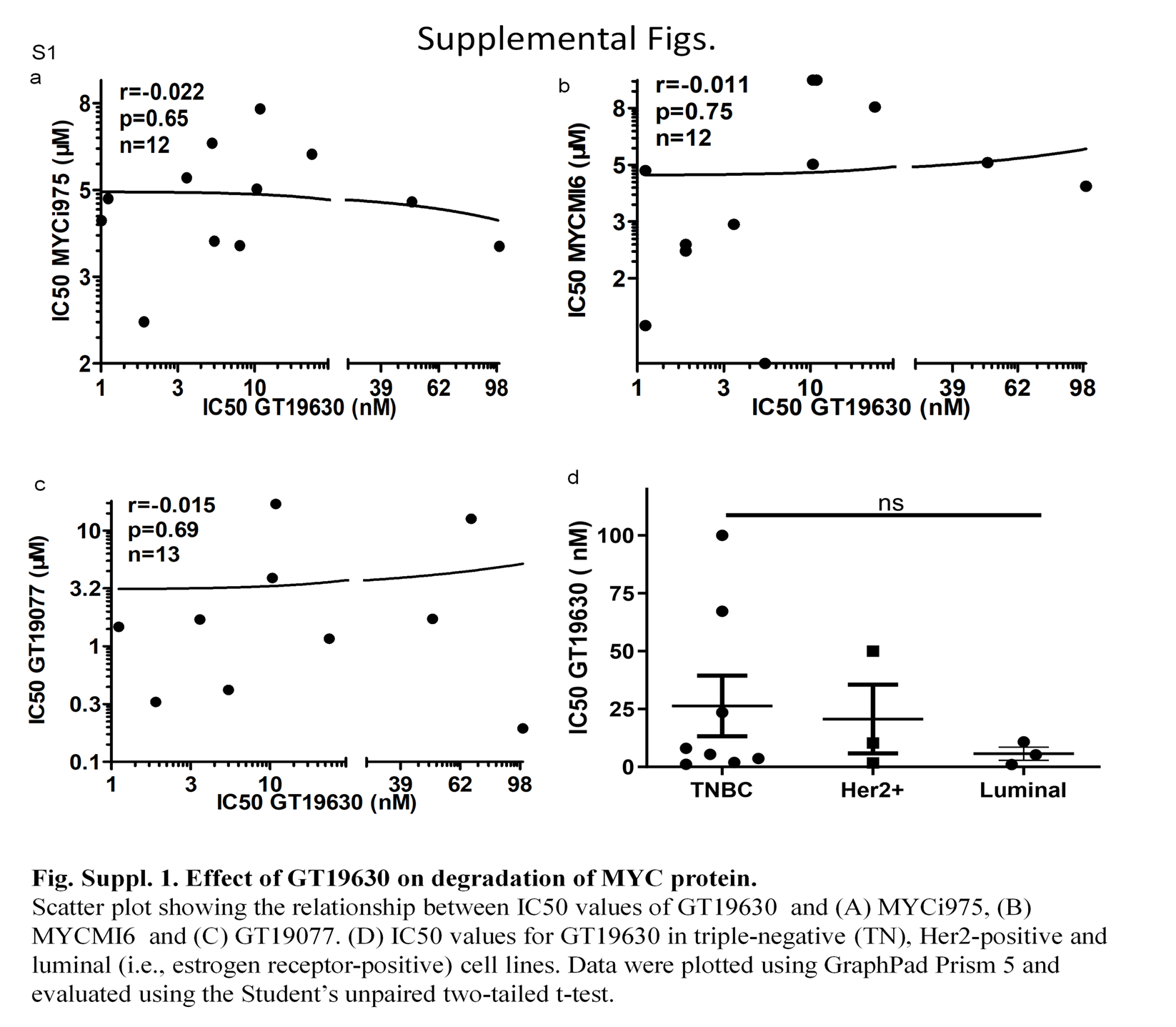

Supplement: Supplementary file 1 — (PNG 264 KB ) [file 10637_2024_1504_Fig8_ESM.png]

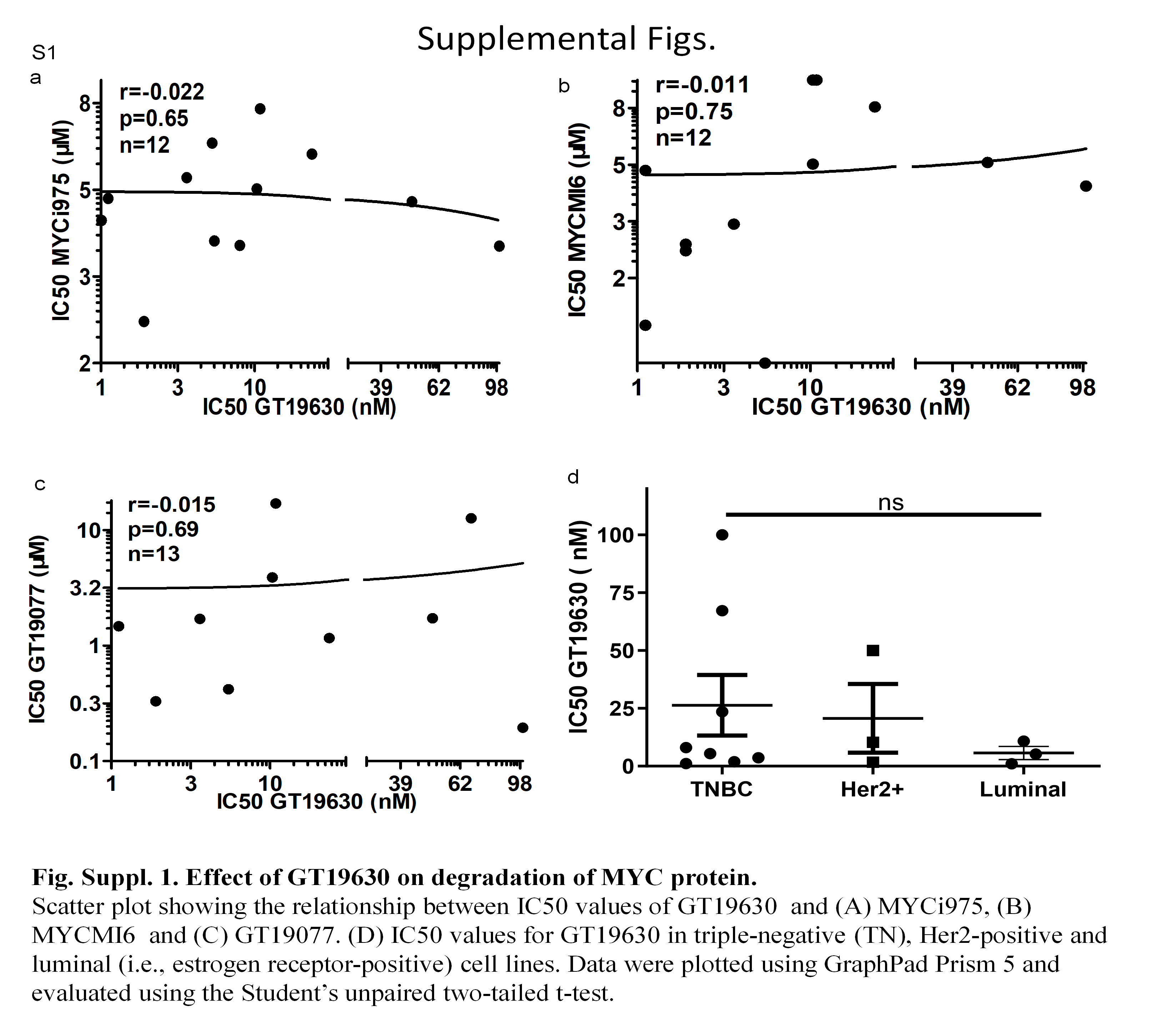

Supplement: Supplementary file 2 — Supplementary file1 (TIF 291 KB) [file 10637_2024_1504_MOESM1_ESM.tif]

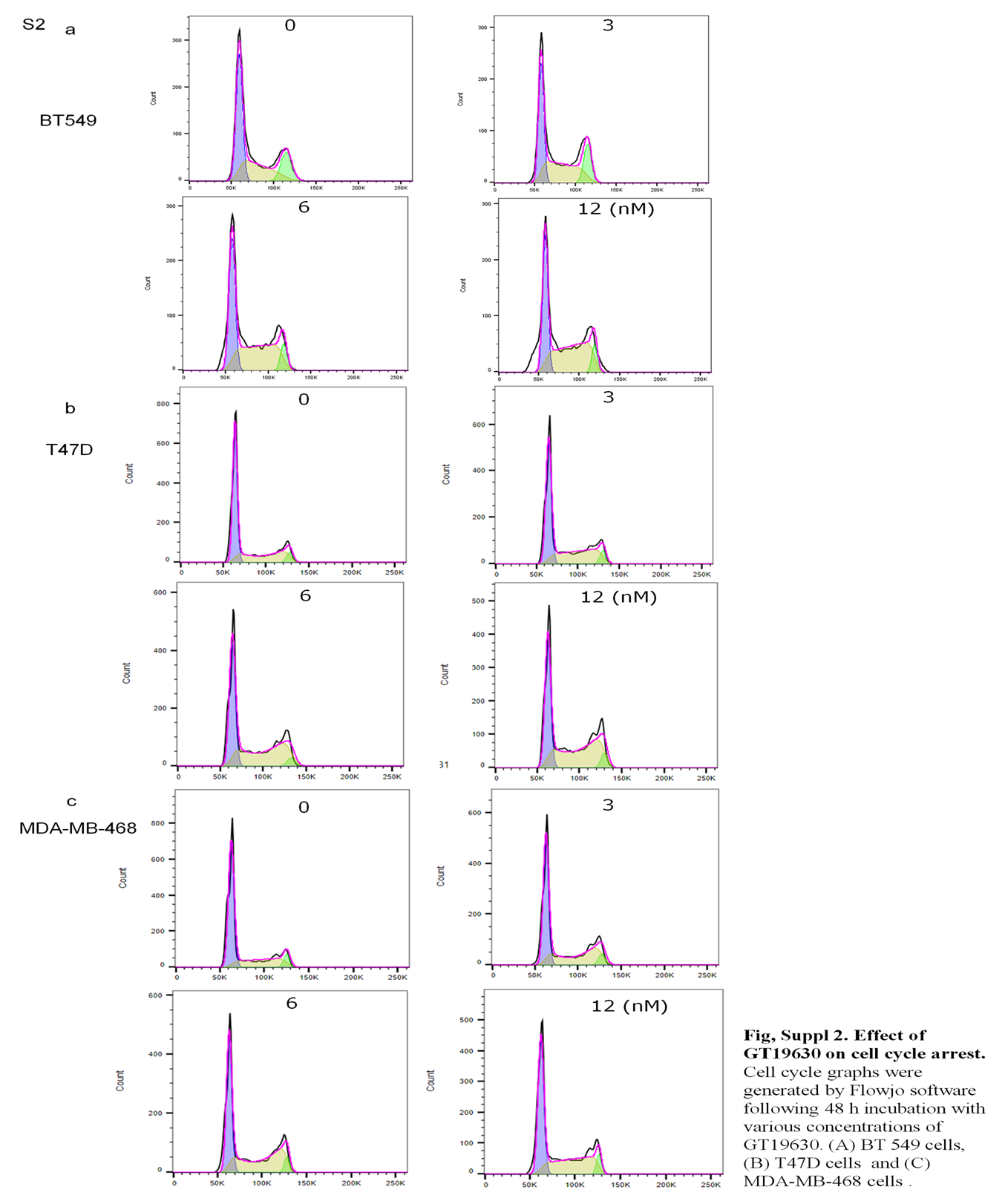

Supplement: Supplementary file 3 — (PNG 285 KB ) [file 10637_2024_1504_Fig9_ESM.png]

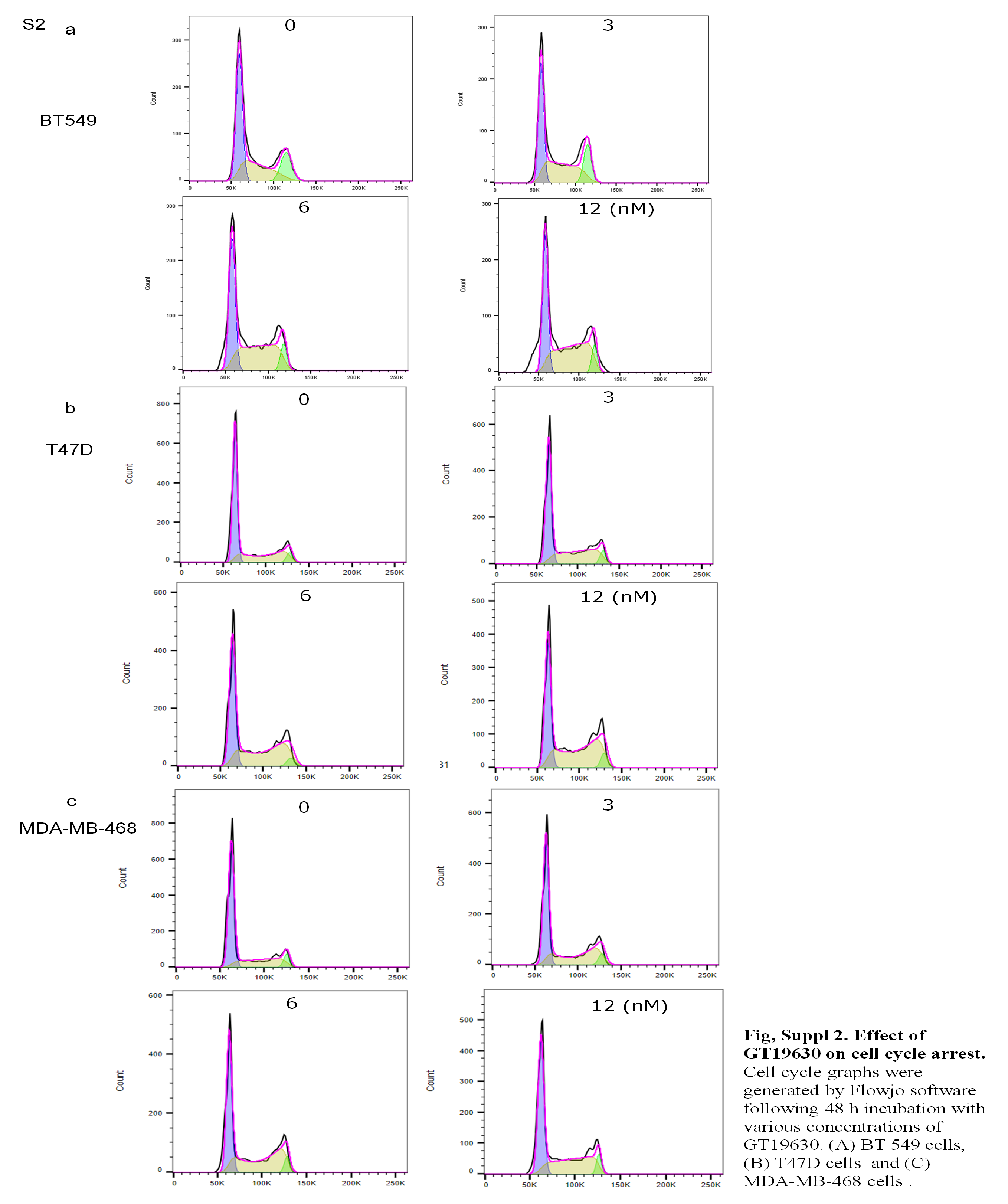

Supplement: Supplementary file 4 — Supplementary file2 (TIF 1040 KB) [file 10637_2024_1504_MOESM2_ESM.tif]

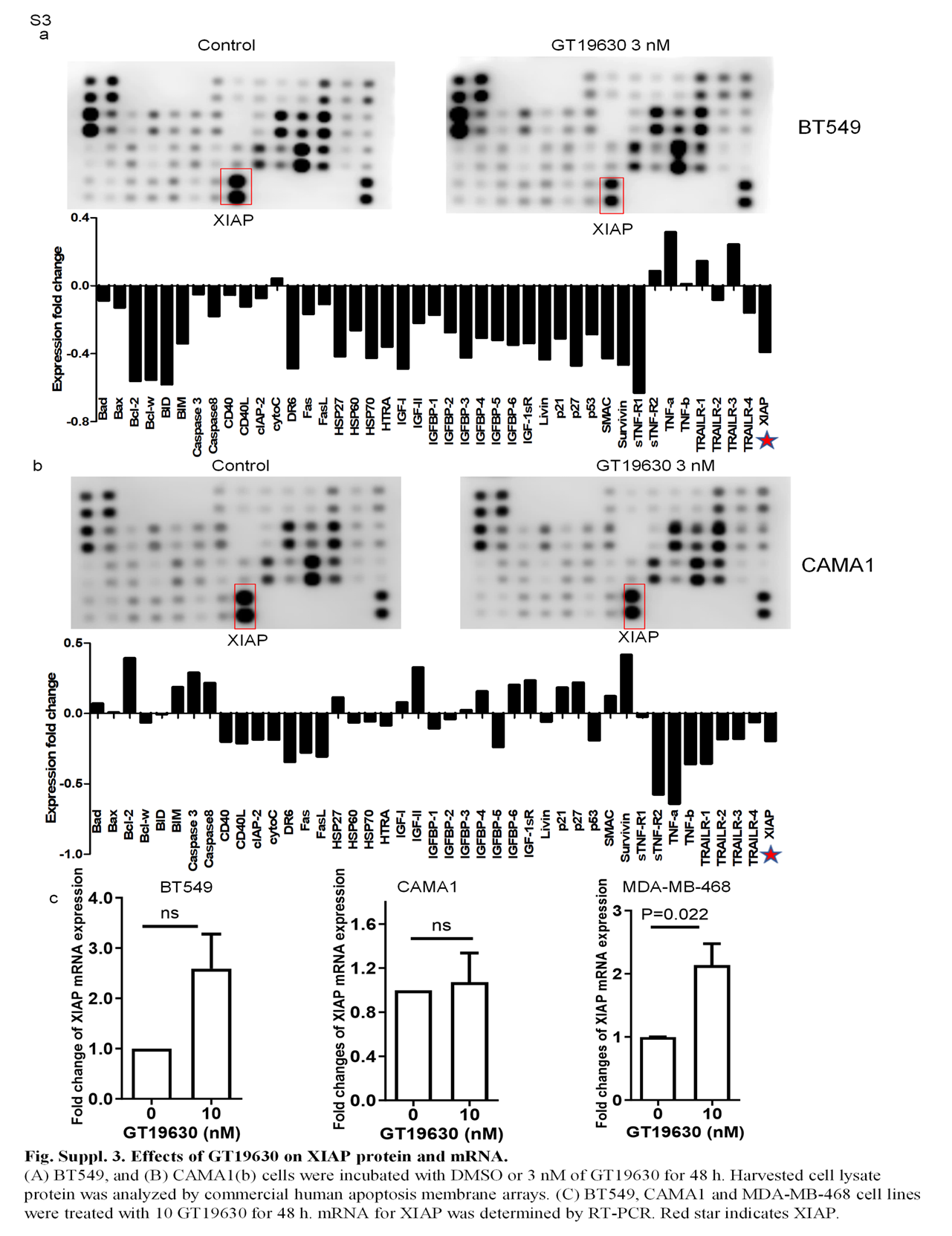

Supplement: Supplementary file 5 — (PNG 463 KB ) [file 10637_2024_1504_Fig10_ESM.png]

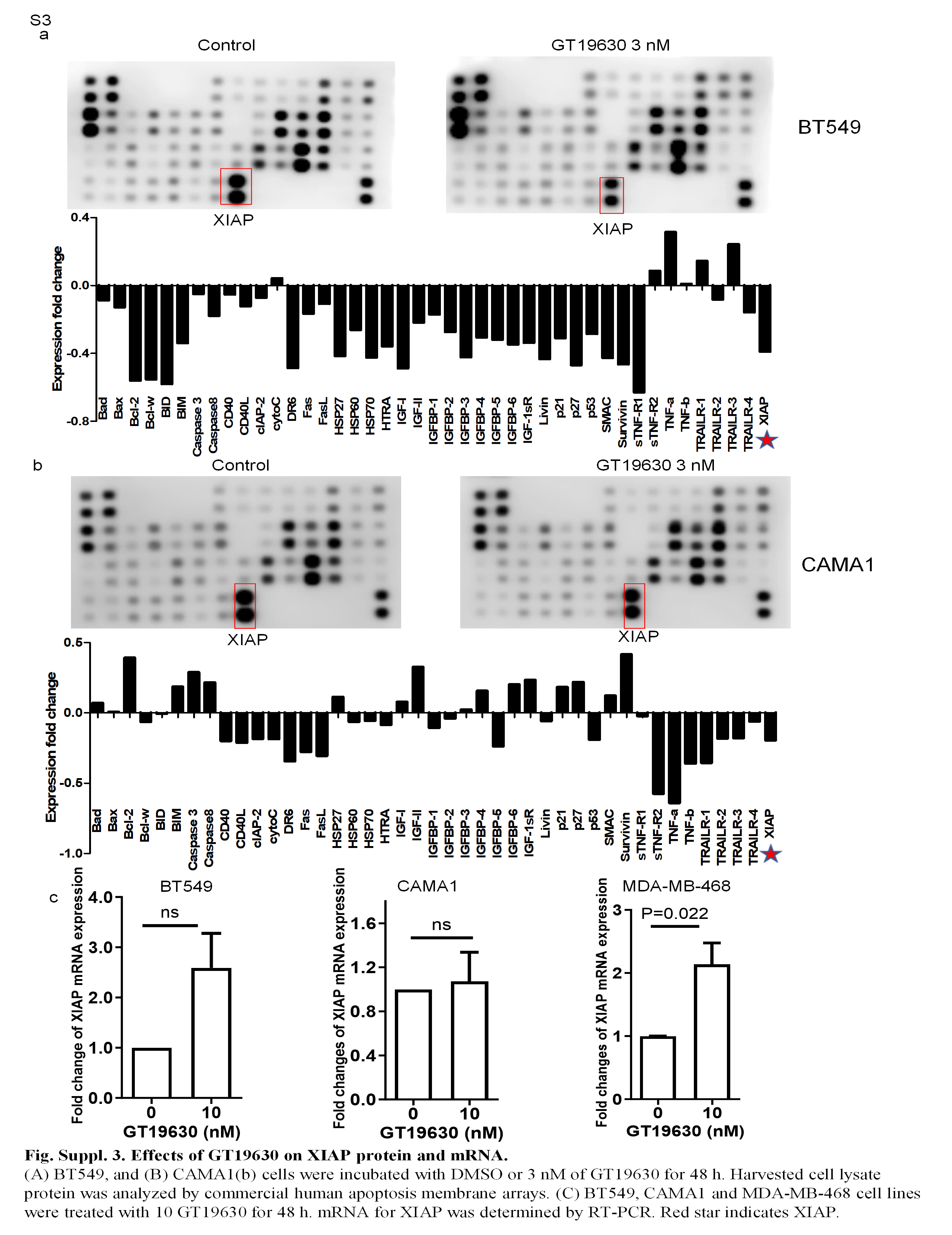

Supplement: Supplementary file 6 — Supplementary file3 (TIF 1509 KB) [file 10637_2024_1504_MOESM3_ESM.tif]

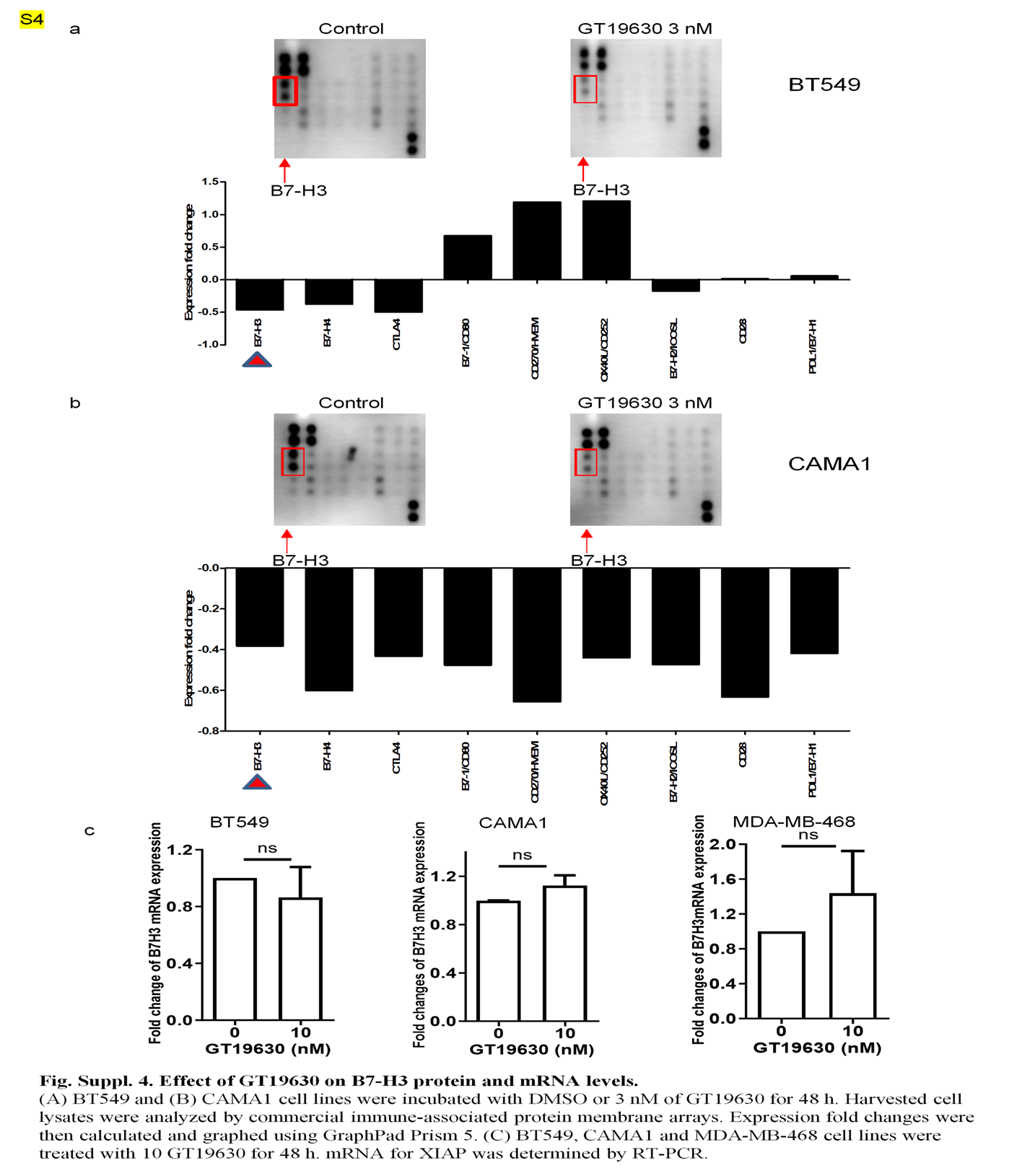

Supplement: Supplementary file 7 — (PNG 307 KB ) [file 10637_2024_1504_Fig11_ESM.png]

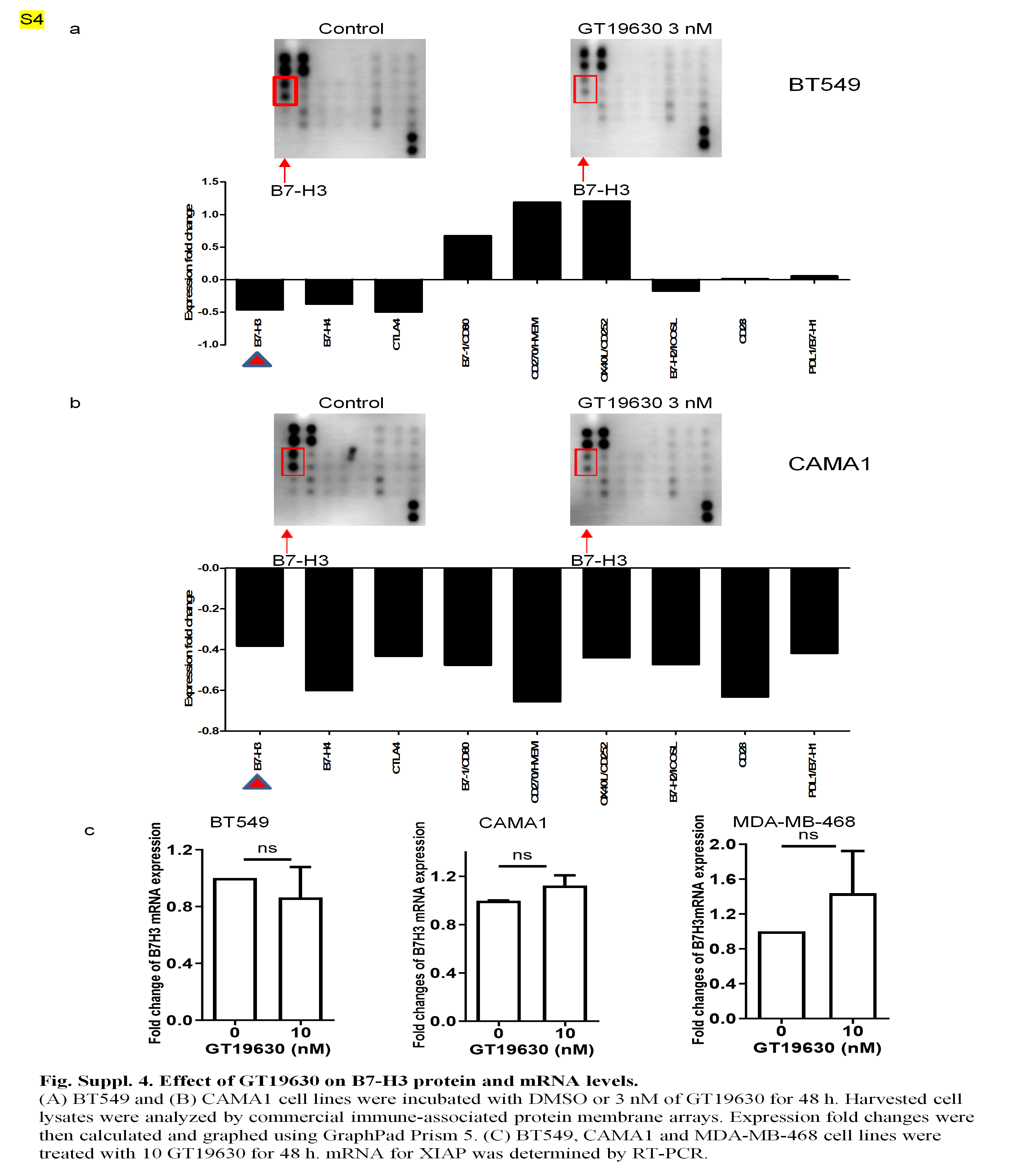

Supplement: Supplementary file 8 — Supplementary file4 (TIF 985 KB) [file 10637_2024_1504_MOESM4_ESM.tif]

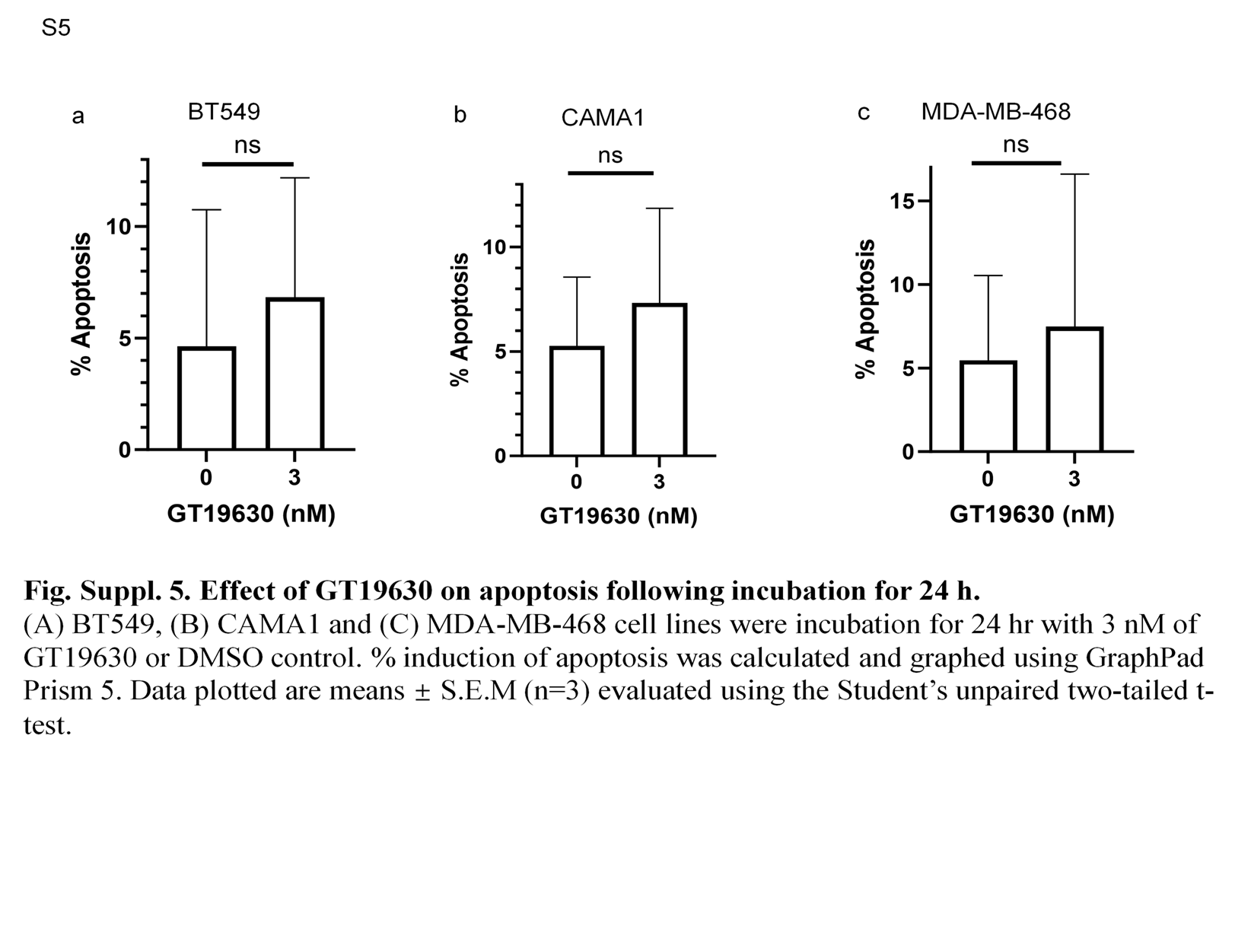

Supplement: Supplementary file 9 — (PNG 140 KB ) [file 10637_2024_1504_Fig12_ESM.png]

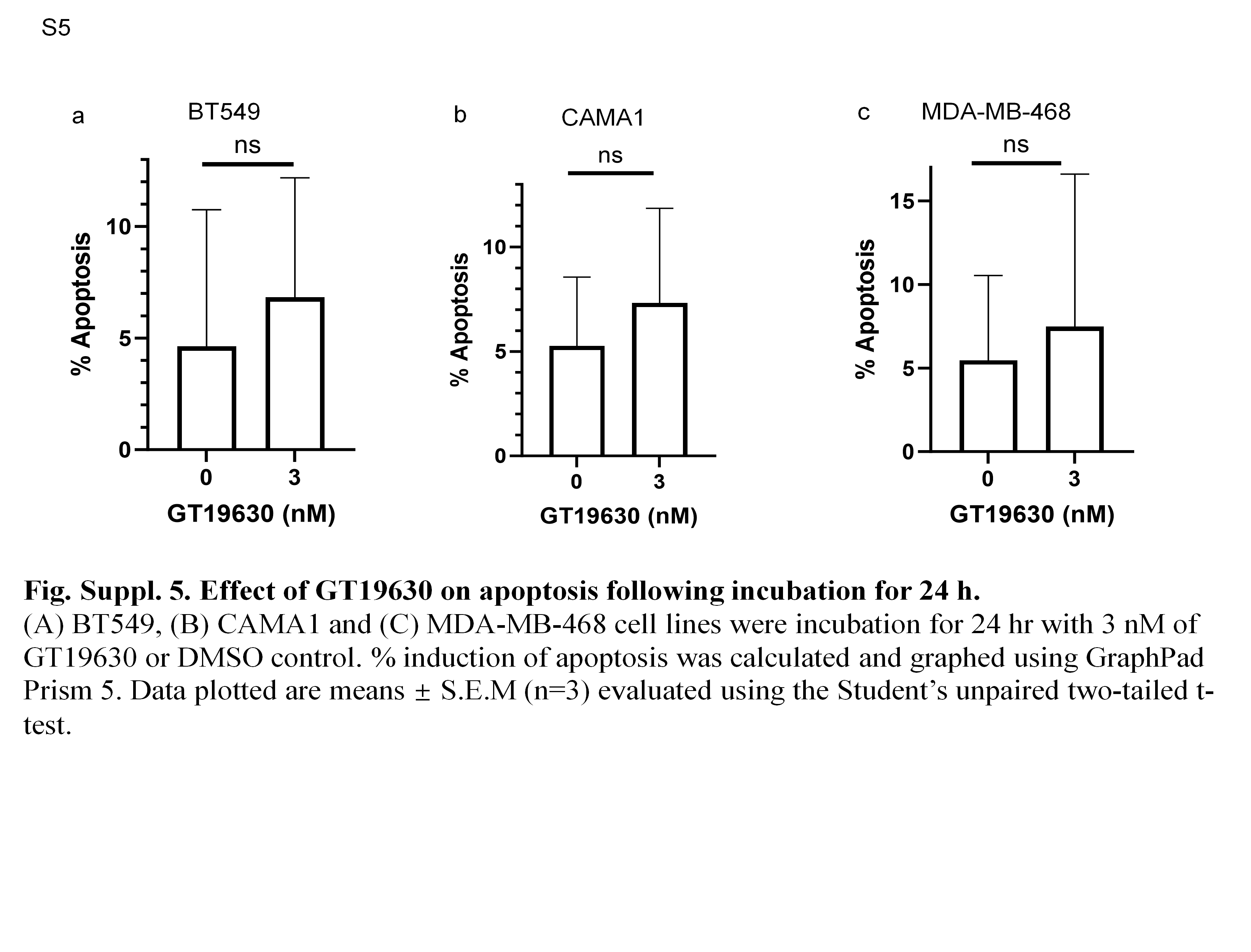

Supplement: Supplementary file 10 — Supplementary file5 (TIF 234 KB) [file 10637_2024_1504_MOESM5_ESM.tif]
